# Activated Microglia Induce Bone Marrow Mesenchymal Stem Cells to Produce Glial Cell-Derived Neurotrophic Factor and Protect Neurons Against Oxygen-Glucose Deprivation Injury

**DOI:** 10.3389/fncel.2016.00283

**Published:** 2016-12-16

**Authors:** Bingke Lv, Feng Li, Jie Fang, Limin Xu, Chengmei Sun, Jianbang Han, Tian Hua, Zhongfei Zhang, Zhiming Feng, Qinghua Wang, Xiaodan Jiang

**Affiliations:** Department of Neurosurgery, Zhujiang Hospital, Southern Medical University, The National Key Clinical Specialty, The Engineering Technology Research Center of Education Ministry of China, Guangdong Provincial Key Laboratory on Brain Function Repair and RegenerationGuangzhou, China

**Keywords:** neuron, bone marrow mesenchymal stem cell, microglia, glial cell-derived neurotrophic factor, tumor necrosis factor, oxygen-glucose deprivation

## Abstract

In this study, we investigated interactions among microglia (MG), bone marrow mesenchymal stem cells (BMSCs) and neurons in cerebral ischemia and the potential mechanisms using an *in vitro* oxygen-glucose deprivation (OGD) model. Rat BMSCs were incubated with conditioned medium (CM) from *in vitro* cultures of OGD-activated rat MG and murine BV2 MG cells. Effects of glial cell-derived neurotrophic factor (GDNF) on rat neuron viability, apoptosis, lactate dehydrogenase (LDH) leakage and mitochondrial membrane potential (MMP) were analyzed in this model. OGD-activated MG promoted GDNF production by BMSCs (*P* < 0.01). Tumor necrosis factor-α (TNFα), but not interleukin-6 (IL6) or interleukin 1β (IL1β), promoted GDNF production by BMSCs (*P* < 0.001). GDNF or CM pre-treated BMSCs elevated neuronal viability and suppressed apoptosis (*P* < 0.05 or *P* < 0.01); these effects were inhibited by the RET antibody. GDNF activated MEK/ERK and phosphoinositide-3-kinase (PI3K)/AKT signaling but not JNK/c-JUN. Furthermore, GDNF upregulated B cell lymphoma 2 (BCL2) and heat shock 60 kDa protein 1 (HSP60) levels, suppressed LDH leakage, and promoted MMP. Thus, activated MG produce TNFα to stimulate GDNF production by BMSCs, which prevents and repairs OGD-induced neuronal injury, possibly via regulating MEK/ERK and PI3K/AKT signaling. These findings will facilitate the prevention and treatment of neuronal injury by cerebral ischemia.

## Introduction

Cerebral ischemia is a condition caused by insufficient blood flow to the brain, which deprives the brain of oxygen, glucose and other essential substrates, and further blocks the normal brain metabolism (Busl and Greer, 2010). Cerebral ischemia is related to the pathogenesis of various diseases such as stroke, hypoxic-ischemic encephalopathy and Alzheimer’s disease (Moroney et al., 1997; Koistinaho and Koistinaho, 2005; Perlman, 2006). The lack of biochemical energy associated with cerebral ischemia induces neuronal injury, which is characterized by accelerated neuronal necrosis and apoptosis (Perlman, 2006). Elucidation of the mechanism underlying cerebral ischemia has indicated several key factors and substances as targets for the treatment of related diseases (Lim et al., 2011; Yang et al., 2013). However, cerebral ischemia remains a complex problem that is yet to be solved.

Bone marrow mesenchymal stem cells (BMSCs), or mesenchymal stem cells, are isolated from bone marrow and possess the capacity to differentiate into various cells types, including osteoblasts, cardiomyocytes, neurons and astrocytes (Montzka et al., 2009; Nandy et al., 2014). Due to these characteristics, BMSCs are showing promise in clinical trials of osteonecrosis and ischemic cardiomyopathy (Hare et al., 2012; Zhao et al., 2012). *In vitro* studies also indicate the anti-inflammatory and anti-immune properties of BMSCs, with great potential in preventing transplant rejection (Franquesa et al., 2012). BMSC treatment inhibits tumor necrosis factor-α (TNFα) production by activated microglia (MG; Kim et al., 2009). Moreover, BMSCs have been reported to prevent neuron cell-like apoptosis (Mo et al., 2012).

MG cells are macrophages in the brain that provide support, nutrition, protection and repair for neurons. These cells are activated by neurotoxic agents or signals released by injured neurons, a process that is often accompanied by elevated MG cells production of TNFα and interleukins, which contribute to the protection or degeneration of neurons (Eskes et al., 2003; Suzuki et al., 2004; Floden et al., 2005). The binary effects of MG on neurons are still under investigation.

Numerous studies have revealed that glial cell-derived neurotrophic factor (GDNF) exerts neuroprotective functions during cerebral ischemia (Lu et al., 2005) and neurotoxicity (Ortiz-Ortiz et al., 2011); thus, GDNF may hold a pivotal position in the interaction between MG and neurons. This study aimed to reveal the interactions between MG and BMSCs in protecting neurons during ischemia injury. MG, BMSCs and hippocampal neurons were isolated from Wistar rats and cultured *in vitro*. MG or the immortalized murine MG cell line BV2 were activated by oxygen-glucose deprivation (OGD), and the conditioned medium (CM) was added to BMSCs to promote GDNF production. The effects of GDNF on OGD-injured neurons were analyzed in cell viability and apoptosis assays. Potential mechanisms were investigated by Western blotting, lactate dehydrogenase (LDH) assays and mitochondrial membrane potential (MMP) detection. These results will provide an improved understanding of the mechanisms underlying neuron protection as well as fundamental information for the development of strategies to prevent and treat cerebral ischemia.

## Materials and Methods

### Animals

BMSCs, MG and neurons were isolated from specific pathogen-free grade Wistar rats (Vital River Laboratories, Beijing, China). The rats were maintained under laboratory conditions with free access to standard diet, sterile water and controlled temperature (24°C). The rats were anesthetized by intraperitoneal injection of chloral hydrate (350 mg/kg) and then euthanized by cervical dislocation for sampling.

All the animal experiments performed in this study were approved by the Animal Ethics Committee of Southern Medical University and were conducted in accordance with the instruction of our institute. No minors, persons with disabilities or endangered animal species were involved.

### Cell Isolation and Culture

BMSCs were isolated from bilateral femurs and tibias of Wistar rats (aged 4 weeks) based on a previously described method (Huang et al., 2015). Briefly, both ends of the bones were removed to expose the marrow cavity, which was washed in serum-free Dulbecco’s modified Eagle medium (DMEM, Gibco, Carlsbad, CA, USA) and the fluid was collected. Cells were collected by centrifugation at 400× g for 10 min, suspended in DMEM supplemented with 10% fetal bovine serum (FBS) and penicillin-streptomycin (100 U/mL, Gibco) at a density of 2 × 10^6^ cells/mL. Cells were then cultured at 37°C in humidified atmosphere under 5% CO_2_. After 3 days, the non-adherent cells were discarded. The culture medium was changed every 3 days and the cells were passaged at approximately 60% confluence.

The immortalized murine MG cell line BV2 (ATCC, Manassas, VA, USA) was cultured in DMEM supplemented with 10% FBS at a density of 1 × 10^5^ cells/mL, and incubated at 37°C in humidified atmosphere under 5% CO_2_.

MG were isolated from the brain of newborn (within 24 h) Wistar rats using a previously described method (Hide et al., 2002). The cerebral cortex was sampled under sterile conditions and washed in cold phosphate-buffered saline (PBS). The meninx and blood vessels on the surface were carefully removed, and the remaining cerebral cortex was then cut into cubes (approximately 1 mm^3^) in serum-free DMEM before digestion in 0.05% trypsin (Gibco) for 30 min at 37°C. The suspension was centrifuged at 400× g for 10 min after termination of the digestion, and the cells were resuspended at a density of 1 × 10^6^ cells/mL in DMEM supplemented with 10% FBS and penicillin-streptomycin (100 U/mL). The cells were then incubated at 37°C in a humidified atmosphere under 5% CO_2_ and after approximately 2 weeks, the cells were separated into two layers—the lower astrocytes and the upper putative MG. The cells were digested in 0.05% trypsin and the dishes were gently shaken to resuspend the MG. The digestion was terminated and MG were isolated from the cell suspension by centrifugation, and cultured in complete DMEM at a density of 1 × 10^6^ cells/mL.

Neurons were isolated from the hippocampus of newborn Wistar rats. Microvessels were carefully removed, and the hippocampus was then cut into pieces and digested in 0.125% trypsin for 20 min at 37°C. The digestion was terminated and the cells were filtered and seeded at a density of 1 × 10^5^ cells/mL in DMEM supplemented with 10% FBS and penicillin-streptomycin (100 U/mL). The cells were incubated at 37°C in humidified atmosphere under 5% CO_2_.

### Experimental Design

To analyze GDNF secretion by BMSCs, BV2 and MG cells were subjected to OGD for 4 h, and the CM was collected and centrifuged at 300× g for 10 min. BMSCs were seeded in 96-well plates at a density of 1 × 10^4^ cells/well. After incubation for 24 h, the medium was replaced entirely with 200 μL CM; BMSCs subjected to this procedure acted as the BMSC + BV2/MG + OGD group. The CM of BV2/MG without OGD induction was also collected and added to BMSCs as the BMSC + BV2/MG group. The BMSC group did not receive any treatment. After incubation for 24 h, the BMSCs culture supernatant in each group was collected for determination of the GDNF concentration. Complete culture medium was used as the control group.

To analyze GDNF production by BMSCs following treatment with TNFα, interleukin (IL) 6 or interleukin 1β (IL1β), the levels of these cytokines in the CM of the activated BV2 and MG were detected by ELISA (see “ELISAs” Section). In addition, BMSCs were treated with TNFα (PeproTech, Rocky Hill, NJ, USA) at final concentrations of 0, 1, 5, 10, 20 or 50 ng/mL, and interleukin-6 (IL6) or IL1β (PeproTech) to final concentrations of 0, 1, 5, 10, 50 or 100 ng/mL. The cells were cultured for 24 h, and the medium was then collected to determine the GDNF concentration.

For analysis of the effects of GNDF in repairing OGD injury, neurons were seeded in 96-well plates (1 × 10^4^ cells/well) for 24 h, before the addition of GDNF (500 ng/L, PeproTech), or replacement of the medium with BMSCs culture supernatant, GDNF-silenced BMSCs (see “siRNA Transfection” Section), CM pre-treated BMSCs, or CM pre-treated GDNF-silenced BMSCs for 24 h after OGD exposure. Additionally, neurons were treated with TNFα (5 ng/mL), IL6 (5 ng/mL) or IL1β (5 ng/mL, PeproTech) for 24 h after OGD.

For detection of the protective effects of GDNF against OGD injury, neurons were seeded into 96-well plates (1 × 10^4^ cells/well) or 6-well plates (1 × 10^5^ cells/well) for 24 h. GDNF (500 ng/L) and polyclonal anti-RET (2 μg/mL, the GDNF receptor) antibody (Santa Cruz Biotechnologies, Santa Cruz, CA, USA) were added to neurons for 24 h before OGD induction.

### OGD Induction

The OGD model was used to activate BV2 and MG cells as well as to injure BMSC and neurons. In brief, the OGD model was established by exposure of cells cultured in glucose-free medium and to a humidified atmosphere of 95% N_2_ and 5% CO_2_ at 37°C for 4 h (Zhou X. et al., 2014). Cells cultured under normal condition were used as a control group.

### siRNA Transfection

For RNA silencing, 150 nmol/L GDNF siRNA (Santa Cruz Biotechnologies) were transfected into BMSCs using Lipofectamine 3000 reagent (Invitrogen) following the manufacturer’s protocol. After 48 h, cells were collected for use in subsequent experiments.

### ELISAs

GDNF concentrations in culture supernatants were determined using the rat GDNF ELISA Kit (Cusabio, Wuhan, China) according to the manufacturer’s instructions. Briefly, the culture supernatant was collected by centrifugation at 300× g for 15 min, and 100 μL was added to each well of 96-well plates. After incubation for 2 h at 37°C, the supernatant was removed and the plates were incubated sequentially with the biotin- conjugated primary detection antibody followed by the horseradish peroxidase (HRP)-conjugated secondary antibody for 1 h at 37°C; plates were washed three times between steps. After addition of the chromogenic substrate, the plates were incubated in the dark for 30 min at 37°C. After the reaction was stopped, optical density (OD) was immediately detected at 450 nm using the iMark microplate reader (Bio-Rad, Hercules, CA, USA). Concentrations of TNFα, IL6 and IL1β in BV2 and MG culture supernatants were also detected using the corresponding ELISA kit from PeproTech according to the manufacturer’s instructions.

### Cell Viability

Neuron cell viability was evaluated after treatment using the MTT Cell Proliferation Assay Kit (ATCC) according to the manufacturer’s instruction. Briefly, neurons (1 × 10^4^ cells) were seeded in each well of 96-well plates for adherence, and treated for 24 h. MTT Reagent was added and the plates were incubated for 4 h before addition of 100 μL Detergent Reagent to dissolve the purple precipitate. The plates were then incubated in the dark at room temperature for 2 h, and the OD was detected at 570 nm using the iMark microplate reader (Bio-Rad).

### Cell Apoptosis

Cell apoptosis was investigated by flow cytometry after fluorescein isothiocyanate (FITC) and propidium iodide (PI) staining with the Annexin V-FITC Apoptosis Kit (BioVision, Milpitas, CA, USA) according to the manufacturer’s instruction. In brief, 1 × 10^5^ were cells resuspended in 500 μL Binding Buffer for each sample. Annexin V-FITC (5 μL) and PI (5 μL) were added and the cells were incubated at room temperature for 5 min in the dark immediately before flow cytometric analysis (BD Biosciences, San Jose, CA, USA). FITC-positive and PI-negative cells were considered to be apoptotic.

### LDH Assay

LDH release by neurons was detected using the LDH Cytotoxicity Detection Kit (Roche, Basel, Switzerland) according to the manufacturer’s instructions. Neurons (1 × 10^4^ cells/well) were seeded into 96-well plates. OD was detected at 500 nm by the iMark microplate reader (Bio-Rad).

### MMP Detection

MMP of neurons was detected by Rhodamin 123 (Sigma-Aldrich) staining. Neurons (1 × 10^6^ cells) were washed twice in artificial cerebral spinal fluid (ACSF, NaCl 124 mM, KCl 3 mM, NaHCO_3_ 26 mM, NaH_2_PO_4_·2H_2_O 1.24 mM, MgSO_4_·7H_2_O 2 mM, CaCl_2_ 2 mM and glucose 10 mM) before addition of Rhodamine 123 at a final concentration of 5 μg/mL. The cells were incubated at 37°C for 45 min and washed again in ACSF before the fluorescence intensity was detected by flow cytometry.

### Western Blotting

The total protein content of neurons was extracted by M-PER Mammalian Protein Extraction Reagent (Thermo Scientific, Carlsbad, CA, USA) according to the manufacturer’s instruction. Protein samples were separated by sodium dodecyl sulfate-polyacrylamide gel electrophoresis and transferred to polyvinylidene fluoride membranes (Roche). The membranes were blocked in 5% skimmed milk for 2 h at room temperature and then incubated at 4°C overnight in rabbit polyclonal or monoclonal antibodies for the specific detection of B cell lymphoma 2 (BCL2, ab59348, Abcam, Cambridge, UK), heat shock 60 kDa protein 1 (HSP60, ab46798), mitogen-activated protein kinase (MAPK) kinase 1/2 (MAP2K1/2 alias MEK1/2, ab178876), phospho-MEK1/2 (p-MEK1/2, ab194754), MAPK3/1 (alias ERK1/2, ab17942), p-ERK1/2 (ab76299), phosphoinositide-3-kinase (PI3K p85, ab191606), p-PI3K (ab182651), v-akt murine thymoma (pan-AKT, ab8805), p-AKT (ab38449), MAPK8/9/10 (alias JNK, ab208035), p-JNK, jun proto-oncogene (c-JUN, ab32137), p-c-JUN (ab32385) and Actin (ab3280) which was used as an internal control. After washing in PBS, the membranes were incubated with the goat anti-rabbit IgG (HRP-conjugated, ab7090) secondary antibody at room temperature for 1 h. Positive signals were developed by EasyBlot ECL Kit (Sangon Biotech, Shanghai, China) and analyzed with ImageJ 1.49 (National Institutes of Health, Bethesda, MD, USA).

### Statistical Analysis

Data represent the mean ± standard deviation (SD) of five independent experiments. Data were analyzed by SPSS 20 (IBM, New York, NY) using Student’s *t* test or one-way analysis of variance (ANOVA) as appropriate. *P* < 0.05 was considered to indicate statistical significance.

## Results

### GDNF Production by BMSCs is Stimulated by TNF*α* from MG

**Figure 1:**
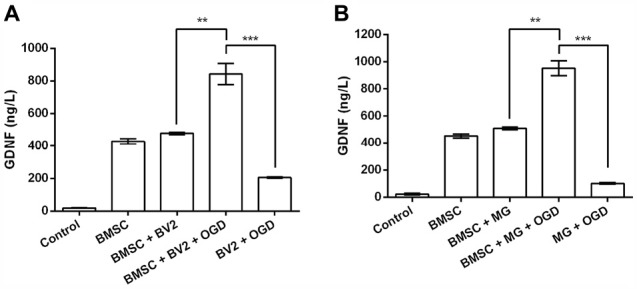
**Conditioned medium (CM) from cultures of oxygen-glucose deprivation (OGD) activated BV2 and microglia (MG) stimulates glial cell-derived neurotrophic factor (GDNF) production by bone marrow mesenchymal stem cells (BMSCs). (A)** BV2 or **(B)** MG cells were exposed to OGD for 4 h before the addition of CM to BMSCs. After 24 h, GDNF concentrations in the BMSC culture supernatants were determined by ELISA. **P < 0.01. ***P < 0.001.

GDNF production by BMSC was confirmed by analysis of GDNF concentrations in the culture supernatants (Figures 1A,B). No obvious changes in GDNF production were detected when BMSCs were treated directly with BV2 or MG CM (*P* > 0.05). However, treatment with the CM of OGD-activated BV2 or MG significantly elevated GDNF levels in BMSCs culture supernatants (*P* < 0.01). Although BV2 and MG also produced GDNF after OGD induction, the levels were significantly lower than that in the BMSC + BV2 + OGD group (*P* < 0.001). It can be speculated that OGD-activated BV2 and MG produce factors that further stimulate GDNF production by BMSCs.

**Figure 2:**
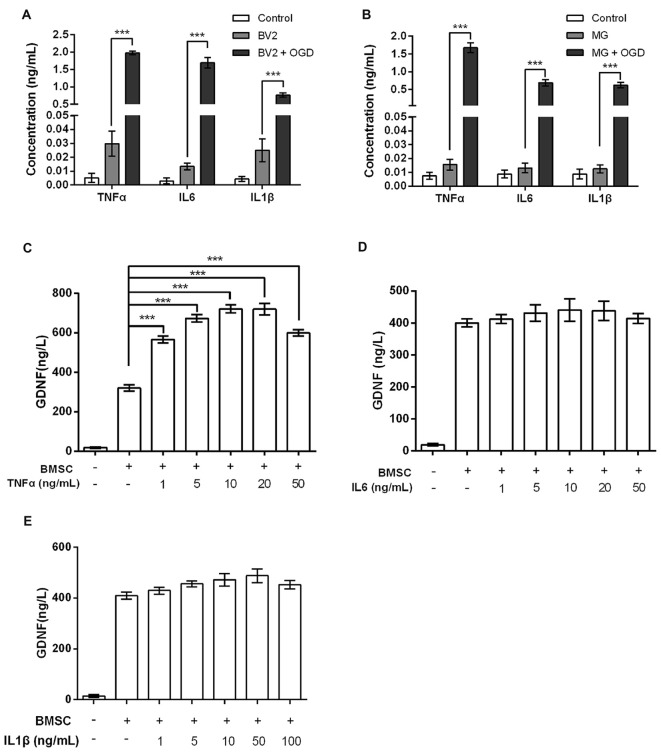
**Tumor necrosis factor-α (TNFα), rather than interleukin-6 (IL6) or interleukin-1β (IL1β), elevates GDNF production by BMSCs.** In **(A)** BV2 and **(B)** MG, OGD stimulates TNFα, IL6 and IL1β production. **(C)** GDNF production by BMSCs is promoted after treatment with TNFα for 24 h. **(D)** IL6 and **(E)** IL1β treatment for 24 h does not significantly change GDNF production by BMSCs. ***P < 0.001.

Numerous studies have shown that MG produce TNFα, IL6 and IL1β in response to OGD exposure (Chock and Giffard, 2005; Zhou et al., 2016); this was also confirmed in our study. The levels of TNFα, IL6 and IL1β produced by BV2 and MG were increased by OGD (*P* < 0.001, Figures 2A,B). Next, we investigated GDNF concentrations in BMSC culture supernatants after treatment with TNFα, IL6 or IL1β. Results showed that BMSCs produced significantly more GDNF after treatment with TNFα (*P* < 0.001, Figure 2C). However, treatment with IL6 and IL1β did not cause an obvious increase in GDNF production (*P* > 0.05, Figures 2D,E), suggesting that TNFα, but not IL6 and IL1β, is secreted by OGD-activated MG, resulting in the induction of GDNF production by BMSCs.

### GDNF Prevents and Repairs OGD-Induced Neuronal Injury

**Figure 3:**
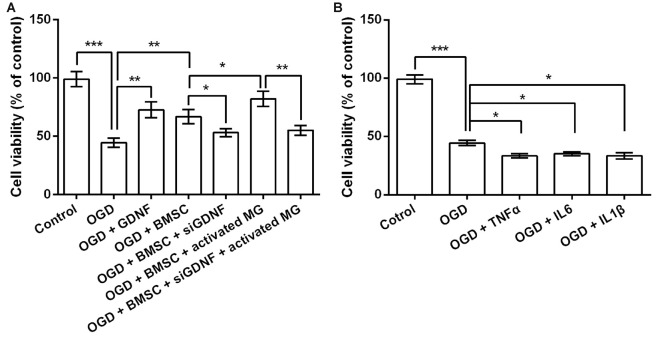
**GDNF repairs OGD-induced neuronal injury. (A)** Neuronal viability is promoted by pretreatment with GDNF after OGD, and is inhibited by GDNF-specific siRNA transfection. **(B)** TNFα, IL6 or IL1β suppresses neuronal viability. *P < 0.05. **P < 0.01. ***P < 0.001.

Next, we performed siRNA-mediated GDNF silencing in BMSCs to assess the functions of GDNF secreted by BMSCs on OGD-injured neurons. As shown in Figure 3A, OGD significantly reduced neuronal viability (*P* < 0.001). Neurons cultured with GDNF or BMSCs CM alleviated OGD-induced suppression of neuronal viability (*P* < 0.01), while this effect was partly abolished by GDNF silencing (*P* < 0.05). Furthermore, CM pre-treated BMSC exhibited a notable increase in neuronal viability (*P* < 0.05), and this increase was also abolished by GNDF silencing (*P* < 0.01). These preliminary data indicate that GNDF produced by BMSCs protects neurons from OGD-induced damage.

Given that OGD induces the production of TNFα, IL6 and IL1β by MG, it is possible that these factors in the CM help to promote neuronal viability via a GDNF-independent mechanism. Therefore, we treated OGD-injured neurons with TNFα, IL6 and IL1β to investigate their effects on cell viability. As shown in Figure 3B, TNFα, IL6 and IL1β treatment diminished neuronal viability (*P* < 0.05), indicating the suppressive role of these pro-inflammatory factors on neuronal viability. Thus, we inferred neuronal viability was promoted mainly by the increased GDNF production by BMSCs, rather than by the TNFα, IL6 and IL1β produced by MG.

**Figure 4:**
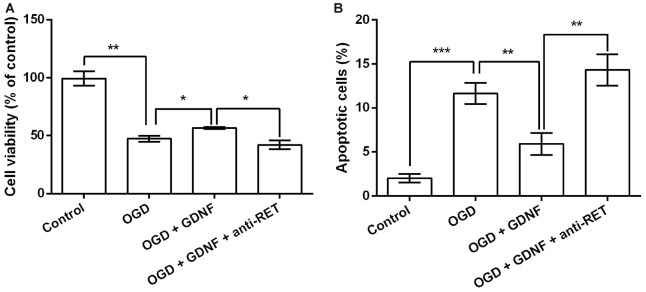
**GDNF prevents OGD-induced neuronal injury. (A)** Neuronal viability is promoted by pretreatment with GDNF before OGD, but is suppressed by anti-RET antibody treatment. **(B)** Neuronal apoptosis is reduced by GDNF pretreatment before OGD, but is improved by anti-RET antibody treatment. *P < 0.05. **P < 0.01. ***P < 0.001.

Neuronal viability and apoptosis were then investigated following treatment with GDNF and the anti-RET antibody that blocks GDNF binding to its receptor before OGD exposure. GNDF prevented the decrease in neuronal viability and the increase in the rate of apoptotic cells rate induced by OGD (*P* < 0.05 or *P* < 0.01, Figures 4A,B). More importantly, these functions of GNDF on neurons were abolished in the presence of the anti-RET antibody (*P* < 0.05 or *P* < 0.01). These data indicated that GNDF prevents OGD-induced neuronal injury.

### GDNF Regulates MEK/ERK, PI3K/AKT and Mitochondrial Stability in Neurons

**Figure 5:**
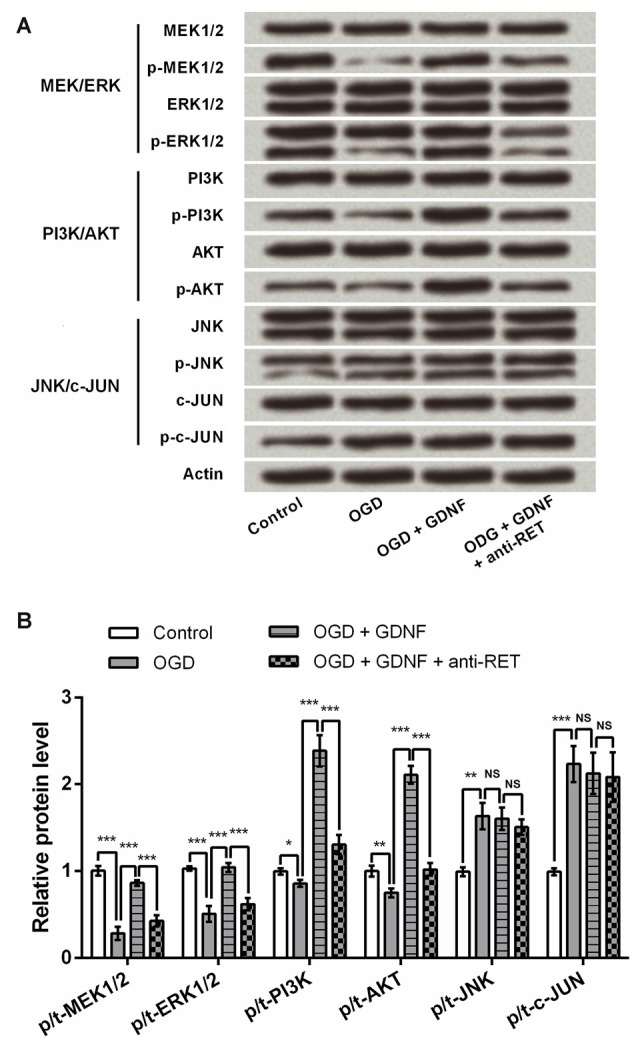
**GDNF regulates the mitogen-activated protein kinase (MAPK) kinase (MEK)/ERK and phosphoinositide-3-kinase (PI3K)/v-akt murine thymoma viral oncogene homolog (AKT) signaling pathways, but not the JNK/c-JUN pathway.** Actin is used as an internal control. **(A)** The phosphorylated forms of MEK1/2, ERK1/2, PI3K and AKT (p-MEK1/2, p-ERK1/2, p-PI3K and p-AKT) are promoted by GDNF and suppressed by anti-RET antibody treatment. The phosphorylated forms of JNK and c-JUN (p-JNK or p-c-JUN) are barely changed by GDNF or anti-RET antibody treatment. **(B)** Relative protein levels based on Western blot results. NS, not significant. *P < 0.05. **P < 0.01. ***P < 0.001.

Previous research has emphasized the involvement of the MEK/ERK, PI3K/AKT and JNK/c-JUN signaling pathways in modulation of cell viability and apoptosis (Oufkir et al., 2010; Park et al., 2012; Dillon et al., 2015); thus, key components of these pathways were detected by Western blotting to investigate the capacity of GDNF to regulate these factors. Results showed that GDNF elevated the expression of p-MEK1/2, p-ERK1/2, p-PI3K and p-AKT, and this GDNF-induced elevation was inhibited in the presence of anti-RET (*P* < 0.001, Figures 5A,B). The total level of these proteins was almost unchanged, suggesting that GDNF promotes their activation. However, p-JNK and p-c-JUN were induced by OGD but were unaffected by GDNF or anti-RET (*P* > 0.05), implying that GDNF regulates activation of the MEK/ERK and PI3K/AKT pathways but not the JNK/c-JUN pathway.

**Figure 6:**
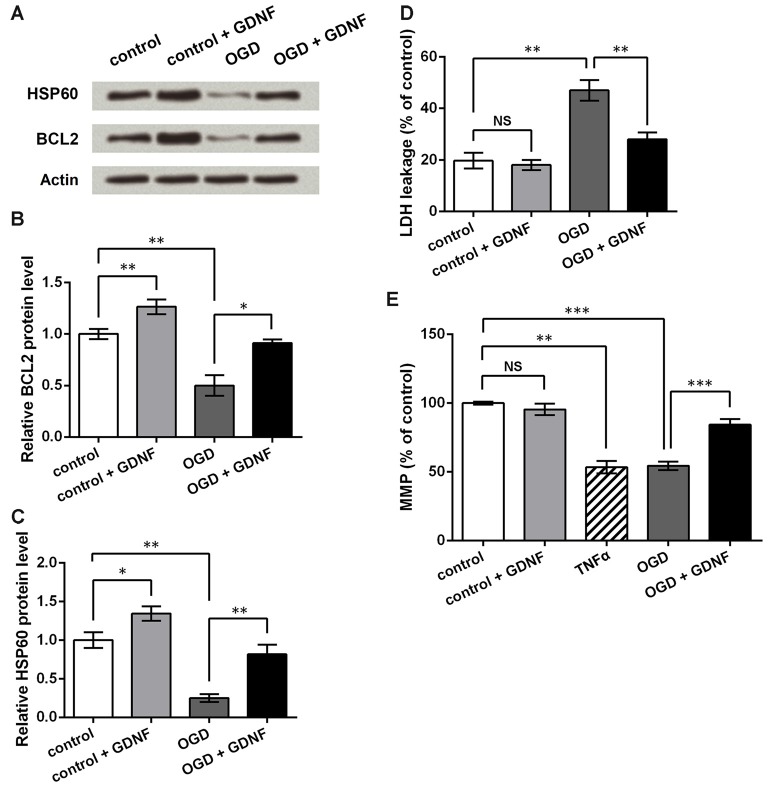
**GDNF helps to maintain mitochondrial membrane potential (MMP) and inhibit neuronal injury induced by OGD. (A)** Western blot showing GDNF up-regulates B cell lymphoma 2 (BCL2) and heat shock 60 kDa protein 1 (HSP60) protein levels in neurons after OGD. Actin is used as an internal control. **(B)** Relative BCL2 protein level based on Western blot results. **(C)** Relative HSP60 protein level based on Western blot results. **(D)** Lactate dehydrogenase (LDH) assay indicates that GDNF inhibits neuronal LDH leakage after OGD. **(E)** MMP assay indicates that GDNF helps to maintain neuronal MMP after OGD, but TNFα decreases MMP. NS, not significant. *P < 0.05. **P < 0.01. ***P < 0.001.

To investigate the potential mechanism by which GDNF attenuates neuronal injury, we also detected the expression of the apoptosis-related protein BCL2 on mitochondrial membrane (Gross et al., 1999), and the molecular chaperone HSP60, which participates in the folding of mitochondrial precursor proteins (Ostermann et al., 1989). Western blot (Figures 6A–C) analysis showed that both BCL2 and HSP60 were downregulated after OGD induction (*P* < 0.01), and were upregulated by GNDF administration with or without exposure to OGD conditions (*P* < 0.05 or *P* < 0.01).

LDH assays showed that OGD elevated LDH leakage from neurons (*P* < 0.01, Figure 6D). Although GDNF had no obvious effects on normal neurons (*P* > 0.05), it markedly decreased LDH leakage in neurons exposed to OGD (*P* < 0.01). Thus, it can be speculated that GDNF helps to maintain the neuronal membrane integrity after OGD. MMP assays showed similar changes in that GDNF significantly elevated MMP after OGD (*P* < 0.001, Figure 6E). In contrast, TNFα treatment of normal neurons led to an apparent decrease in MMP (*P* < 0.01), further suggesting that the effects mediated by TNFα on neurons are opposite to those mediated by GDNF. These results indicate that GDNF maintains mitochondrial stability and suppresses neuronal injury, thus representing a potential mechanism by which GDNF protects against OGD-induced neuronal injury.

## Discussion

Inspired by existing studies demonstrating the protective roles of MG and BMSCs on neurons, we constructed an *in vitro* OGD model in cultured rat neurons, which were then treated with CM of BMSCs and OGD-activated MG. Activated MG produced factors including TNFα, which are implicated in elevation of GDNF production by BMSCs and further protecting neurons from OGD-induced injury. Furthermore, our findings indicate that the mechanism by GDNF protected neurons from OGD-induced injury is, at least partially, mediated via activation of the MEK/ERK and PI3K/AKT signaling pathways and maintenance of mitochondrial stability.

Analysis of GDNF concentrations in BMSCs culture supernatants by ELISA indicated significant upregulation of GDNF after treatment with the CM of OGD-activated MG. Our results are in accordance with other reports that activated MG, as well as BMSCs, produce GDNF (García et al., 2004; Matsushita et al., 2008; Tanaka et al., 2008; Lin et al., 2015). GDNF production was detected in the culture supernatants of untreated BMSCs and OGD-induced BV2/MG. However, GDNF levels were further upregulated when BMSCs were cultured in the CM of OGD-induced BV2/MG. Thus, it can be deduced that OGD-induced MG secrete factors that facilitate GDNF production by BMSCs.

TNFα, IL6 and IL1β are pro-inflammatory factors secreted by MG activated by stimuli such as OGD (Badiola et al., 2009; Ziemka-Nałęcz et al., 2013). Our results showed that TNFα (1–50 ng/mL) promoted GDNF production by BMSCs, while IL6 and IL1β had no significant effects. TNFα exposure has been found to promote proliferation and invasion by BMSCs (Miettinen et al., 2011), without affecting pluripotency (Zhang Z. et al., 2010; Lee et al., 2012), while IL1β and IL6 treatment inhibits chondrocytic differentiation or maintains the stemness of BMSCs (Pricola et al., 2009; Ferreira et al., 2013). Thus, we speculate that the functional disparity between TNFα, IL6 and IL1β in BMSCs may be related to the differences in the signaling pathways regulated by these cytokines. The promoting role of TNFα on GDNF production, as well as the elevated GDNF production by OGD-induced MG, suggests that TNFα is one of the factors produced by MG that stimulates GDNF production by BMSCs.

OGD, which is a common method to construct *in vitro* models for ischemia research, leads to forms of injury including neuronal necrosis and apoptosis (Pei and Cheung, 2003; Chung et al., 2007), whereas GDNF exerts a protective effect on neurons, stimulating viability, proliferation and survival (Saavedra et al., 2006; Han et al., 2015). In accordance with previous reports, we demonstrated that OGD decreased neuronal viability and increased apoptosis, which might reflect neuronal injury caused by this treatment. Both pre and post-treatment with GDNF promoted a decrease in neuronal viability and reduced the number of apoptotic neurons to some extent, suggesting that GDNF prevents and repairs OGD-induced neuronal injury. Since GDNF production by BMSCs is further upregulated by factors such as TNFα, BMSCs may play a pivotal role in the protection of neurons via the functions of GDNF. We cannot exclude the possibility that BMSCs are stimulated to produce factors in addition to GDNF in this process, since multiple mechanisms have been discovered to be involved in the protective effects of BMSCs on neurons (Hokari et al., 2008). However, our results refute the possibility that neuronal viability is increased by TNFα, IL6 and IL1β produced by activated MG, because these factors had obvious negative effects on viability. Elucidation of the precise mechanism requires further investigation of the altered factors in culture supernatants.

**Figure 7:**
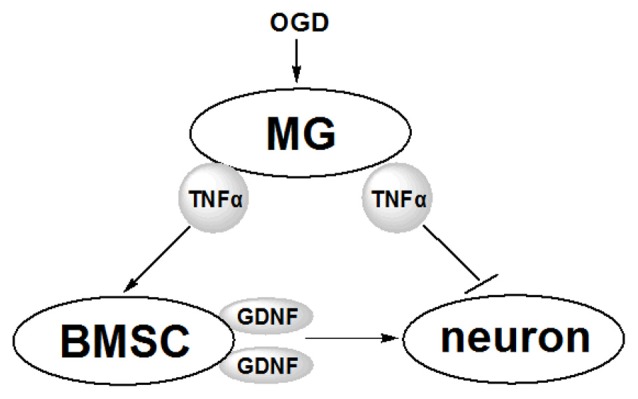
**Schematic diagram of the effects of MG and BMSC on neurons revealed in this study.** Following the stimulation by OGD, MG secretes TNFα, which further induces BMSCs to produce more GDNF. GDNF protects neurons against OGD-induced injury. Furthermore, TNFα secreted from MG induces inflammatory responses to suppress OGD-induced neuronal injury.

TNFα exerts both positive and negative influences on neurons via multiple mechanisms (Saha et al., 2009; Bliss et al., 2011). This study revealed one of the possible mechanism involving the TNFα and GDNF produced by MG and BMSCs, respectively (Figure 7). Following OGD induction, MG produces elevated levels of TNFα that influence neurons in two ways. On the one hand, TNFα suppresses neuron viability directly, possibly via the anabatic inflammatory responses; on the other hand, TNFα induces increased GDNF production by BMSCs to protect neurons. Thus, the protective effects of GDNF on neurons during ischemia may be one of the core links in the interaction between neurons, BMSCs and MG.

Some researchers have detected the inhibitory role of BMSCs on the production of pro-inflammatory factors including TNFα and IL1β by activated MG (Jose et al., 2014; Zhou X. L. et al., 2014). In this study, we investigated the related mechanism from another perspective regarding GDNF and three signaling pathways that regulate neuronal viability and apoptosis. It has been reported that MEK/ERK pathway has neuroprotective functions in primary cultures of rat hippocampal neurons (Wang et al., 2013). PI3K/AKT signaling is necessary for the protection of cortical neurons (Zhang L. et al., 2010), while activation of JNK/c-JUN signaling has been detected in the brain neurons in Alzheimer’s disease models (Akhter et al., 2015). Our results indicate that GDNF regulates these three signaling pathways in a discriminatory manner to promote the activation of MEK/ERK and PI3K/AKT without influencing JNK/c-JUN. Hence, MEK/ERK and PI3K/AKT signaling are implicated in the mechanism by which GDNF prevents and repairs neuronal injury.

In addition to the MEK/ERK and PI3K/AKT signaling pathways, Western blot analysis showed that GDNF also regulated BCL2 and HSP60 expression. Based on the known roles of BCL2 and HSP60 in cell apoptosis and mitochondrial metabolism, the observed upregulation of these factors by GDNF suggests that GDNF protects neurons against OGD-induced injury, possibly by regulating cell apoptosis. We further demonstrated that GDNF suppressed LDH leakage and promoted MMP in the OGD model. Mitochondrial membrane damage and decreased MMP are considered to be early markers of cell apoptosis (Garedew and Moncada, 2010), and increased LDH leakage reflects accelerated cell injury and cell death; thus, these results indicate that mitochondria-related processes are involved in the mechanism by which GDNF protects neurons from OGD-induced injury. Based on research showing that HSP60 up-regulates BCL2 and attenuates cell death (Ghribi et al., 2001; Shan et al., 2003), we speculate that the detailed mechanism of this protection involves the regulation of BCL2 and HSP60 by GDNF, possibly via the mitochondria apoptosis pathway. However, this hypothesis requires further verification.

In conclusion, this study indicates that TNFα secreted by activated MG stimulates BMSCs to produce higher levels of GDNF, which protects against the OGD-induced neuronal injury, possibly by regulating the MEK/ERK and PI3K/AKT signaling pathways. The precise mechanisms involved in the interaction of neurons, BMSCs and MG require further investigation to facilitate the development of strategies for the prevention and treatment of neuron damage by cerebral ischemia.

## Author Contributions

In this study, BL and FL designed the work, drafted the manuscript, conducted the experiments and contributed to interpretation of data. JF, LX, CS, JH and TH conducted the experiments, analyzed the data and helped to prepare the manuscript. ZZ and ZF helped to prepare the manuscript and images, collected and analyzed the data and literature. XJ guided in designing the study and preparing the manuscript, and supported for the research funding. All authors read and approved the final manuscript. All authors agreed to be accountable for all aspects of the study in ensuring that questions related to the accuracy or integrity of any part of the work are appropriately investigated and resolved.

## Funding

This study was supported by grants from the Natural Science Funds of China (No. 81171179, No. 81272439), Funds for Key Natural Science Foundation of Guangdong (No. S2013020012754) and the Educational Commission of Guangdong (No. 2013CXZDA008), Key Projects of Health Collaborative Innovation of Guangzhou and Guangdong (No. 201400000003-2, 2016B030230004) to Prof. Xiaodan Jiang; also part of Funds from Science and Technology Program of Guangdong (No. 2012B031800152, No. 2013B021800158) to Dr. Qinghua Wang; and part of Fund from the Guangdong Provincial Clinical Medical Centre for Neurosurgery (No. 2013B020400005).

## Conflict of Interest Statement

The authors declare that the research was conducted in the absence of any commercial or financial relationships that could be construed as a potential conflict of interest.

## Abbreviations

BCL2: B cell lymphoma 2
BMSCs: bone marrow mesenchymal stem cells
CM: conditioned medium
GDNF: Glial cell-derived neurotrophic factor
HSP60: heat shock 60 kDa protein 1
IL1β: interleukin-1β
IL6: interleukin-6
LDH: lactate dehydrogenase
MG: microglia
MMP: mitochondrial membrane potential
OGD: oxygen-glucose deprivation
TNFα: tumor necrosis factor-α.
